# Variant patterns of electrical activation and recovery in normal human hearts revealed by noninvasive electrocardiographic imaging

**DOI:** 10.1093/europace/euae172

**Published:** 2024-07-05

**Authors:** Job Stoks, Kiran Haresh Kumar Patel, Bianca van Rees, Uyen Chau Nguyen, Casper Mihl, Peter M Deissler, Rachel M A ter Bekke, Ralf Peeters, Johan Vijgen, Paul Dendale, Fu Siong Ng, Matthijs J M Cluitmans, Paul G A Volders

**Affiliations:** Department of Cardiology, Cardiovascular Research Institute Maastricht (CARIM), Maastricht University Medical Center+, Maastricht, The Netherlands; Department of Advanced Computing Sciences, Maastricht University, Maastricht, The Netherlands; Faculty of Medicine and Life Sciences, UHasselt, Diepenbeek, Belgium; Department of Cardiology, Hartcentrum, Jessa Hospital, Hasselt, Belgium; National Heart and Lung Institute (NHLI), Imperial College London, London, UK; Department of Cardiology, Cardiovascular Research Institute Maastricht (CARIM), Maastricht University Medical Center+, Maastricht, The Netherlands; Department of Cardiology, Cardiovascular Research Institute Maastricht (CARIM), Maastricht University Medical Center+, Maastricht, The Netherlands; Department of Cardiology, Cardiovascular Research Institute Maastricht (CARIM), Maastricht University Medical Center+, Maastricht, The Netherlands; Department of Radiology and Nuclear Medicine, Maastricht University Medical Center+, Maastricht, The Netherlands; Department of Cardiology, Cardiovascular Research Institute Maastricht (CARIM), Maastricht University Medical Center+, Maastricht, The Netherlands; Department of Cardiology, Cardiovascular Research Institute Maastricht (CARIM), Maastricht University Medical Center+, Maastricht, The Netherlands; Department of Advanced Computing Sciences, Maastricht University, Maastricht, The Netherlands; Faculty of Medicine and Life Sciences, UHasselt, Diepenbeek, Belgium; Department of Cardiology, Hartcentrum, Jessa Hospital, Hasselt, Belgium; Faculty of Medicine and Life Sciences, UHasselt, Diepenbeek, Belgium; Department of Cardiology, Hartcentrum, Jessa Hospital, Hasselt, Belgium; National Heart and Lung Institute (NHLI), Imperial College London, London, UK; Department of Cardiology, Cardiovascular Research Institute Maastricht (CARIM), Maastricht University Medical Center+, Maastricht, The Netherlands; Department of Cardiology, Cardiovascular Research Institute Maastricht (CARIM), Maastricht University Medical Center+, Maastricht, The Netherlands

**Keywords:** ECGI, Ventricles, Electrophysiology, Variability, Normal, Dynamics

## Abstract

**Aims:**

Although electrical activity of the normal human heart is well characterized by the electrocardiogram, detailed insights into within-subject and between-subject variations of ventricular activation and recovery by noninvasive electroanatomic mapping are lacking. We characterized human epicardial activation and recovery within and between normal subjects using non-invasive electrocardiographic imaging (ECGI) as a basis to better understand pathology.

**Methods and results:**

Epicardial activation and recovery were assessed by ECGI in 22 normal subjects, 4 subjects with bundle branch block (BBB) and 4 with long-QT syndrome (LQTS). We compared characteristics between the ventricles [left ventricle (LV) and right ventricle (RV)], sexes, and age groups (<50/≥50years). Pearson’s correlation coefficient (CC) was used for within-subject and between-subject comparisons. Age of normal subjects averaged 49 ± 14 years, 6/22 were male, and no structural/electrical heart disease was present. The average activation time was longer in LV than in RV, but not different by sex or age. Electrical recovery was similar for the ventricles, but started earlier and was on average shorter in males. Median CCs of between-subject comparisons of the ECG signals, activation, and recovery patterns were 0.61, 0.32, and 0.19, respectively. Within-subject beat-to-beat comparisons yielded higher CCs (0.98, 0.89, and 0.82, respectively). Activation and/or recovery patterns of patients with BBB or LQTS contrasted significantly with those found in the normal population.

**Conclusion:**

Activation and recovery patterns vary profoundly between normal subjects, but are stable individually beat to beat, with a male preponderance to shorter recovery. Individual characterization by ECGI at baseline serves as reference to better understand the emergence, progression, and treatment of electrical heart disease.

## Graphical Abstract

**Graphical Abstract:**
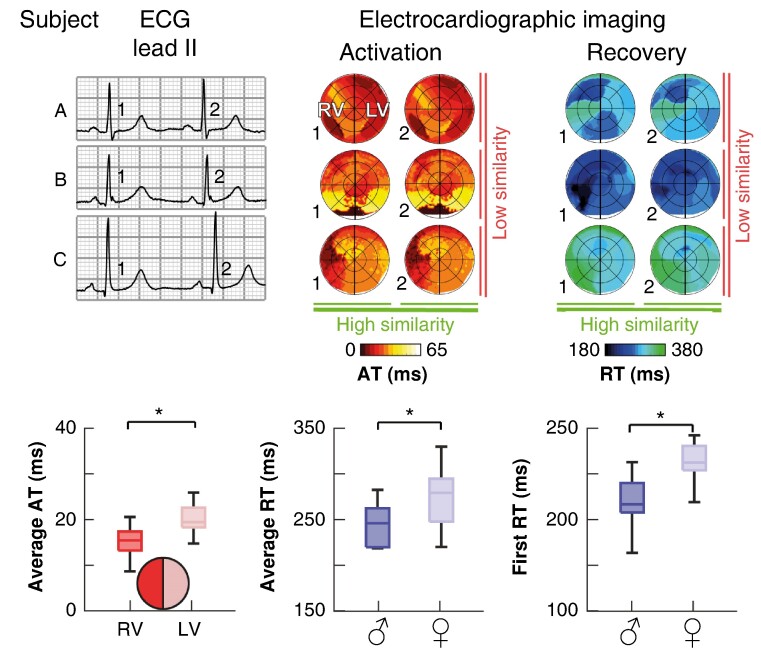
Application of electrocardiographic imaging in healthy controls allows studying normal epicardial activation and recovery patterns. Top: Although all subjects had a normal ECG, we found that their underlying activation time (AT) and recovery time (RT) patterns could be profoundly different. Still, beat-to-beat AT and RT patterns within one subject were relatively similar. Bottom: On a population level, we found that average right ventricular (RV) AT was lower than left ventricular (LV) AT, and both first and average RT were lower in males than in females.

**What’s new?**

- Despite similar unremarkable 12-lead ECGs, ventricular electrical activation, and recovery patterns vary significantly between normal individuals. These differences potentially account for variations in the individual susceptibility to arrhythmias.
- Ventricular electrical activation and recovery are relatively stable over consecutive beats and over several minutes under normal conditions. Dynamic alterations revealed by electrocardiographic imaging may be causal or consequent to arrhythmic perturbations, and owing to the onset/progression of (genetic) cardiac disease.
- Our detailed results of ventricular activation and recovery sequences under physiological conditions provide a basis for better understanding of pathology. In future studies, we aim to investigate the relationship of these individual electrical characteristics at baseline with arrhythmia propensity.

## Introduction

Since the introduction of the 3-lead and 12-lead ECG, insights into the normal activation and recovery of the human heart have increased dramatically. However, the spatial resolution of the 12-lead ECG limits understanding of localized activation or recovery patterns. Catheter mapping provides high resolution, but is invasive and is only performed in (suspected) disease conditions^1,2^ or on explanted hearts.^3–5^

Electrocardiographic imaging (ECGI) enables non-invasive electrical mapping with a ∼1-cm resolution on the ventricular epicardium.^6^ To date, ECGI studies in normal human subjects under physiological conditions are limited to a few only.^7,8^ Detailed insights into the individual characteristics of beat-to-beat dynamics of ECGI-based ventricular electrophysiology and the influence of sex and age are lacking.

We and others have previously validated ECGI (see Supplementary material online, *Figure S1*). To fill current knowledge gaps, we used it to examine beat-to-beat stability of electrical activation and recovery within and among subjects of different ages and compared females and males. Furthermore, to provide a context for electrophysiological variation, we contrasted our results in normal individuals with those of patients with abnormal activation [left bundle branch block (LBBB) and right bundle branch block (RBBB)] or recovery patterns [long-QT syndrome (LQTS)].

## Methods

### Study population and consent

This study was approved by the local ethics committees of Maastricht University Medical Center + (MUMC+), The Netherlands (METC 11-2-043), and the Health Research Authority London, UK (Surrey; 19/LO/0762), and adhered to the Declaration of Helsinki. All subjects provided written informed consent prior to inclusion. Twenty-two normal human subjects were recruited prospectively from the MUMC+, The Netherlands (*n* = 11), and the Imperial College London (ICL), UK (*n* = 11). Inclusion criteria for normal subjects were as follows: age ≥18 years, structurally normal heart on echocardiogram, normal 12-lead ECG, and no (suspected) pathology affecting ventricular electrophysiology. Subjects at the MUMC + underwent a cardiac CT scan as part of routine clinical care (often for atypical chest complaints), ruling out significant coronary artery disease or other cardiac abnormalities. Two subjects received metoprolol (50 or 100 mg) for hypertension or atypical complaints. Subjects at ICL were recruited by advertisement, underwent cardiac MRI, and were likewise negative for pathological findings on echocardiogram, MRI, or ECG.

Eight cardiac patients were recruited by their cardiologist at MUMC+: one with complete RBBB, three with complete LBBB, and four with congenital LQTS. They underwent a cardiac CT scan as part of the study protocol. All LQTS patients had pathogenic mutations in *KCNQ1*, *KCNH2*, or *SCN5A.* Two patients had previous ventricular tachyarrhythmias and an overtly prolonged QTc interval [mutations in the *KCHN2* gene (‘LQT2 Symp.’) and *SCN5A* gene (‘LQT3 Symp.’)]; the other two did not [*KCNQ1-* (‘LQT1 Asymp.’) and *SCN5A*-gene mutation (‘LQT3 Asymp.’)] (see *Table 1*).

**Table 1:** Subject characteristics

Group	ID	Age (y)	Sex	Height (cm)	Weight (kg)	LVEF (%)	QRS (ms)	QTcB (ms)
Normal	1	60	F	169	75	61	88	431
	2	65	F	163	65	61	94	404
	3	65	M	170	70	69	98	398
	4	56	F	177	128	53	92	419
	5	67	F	155	57	55	80	406
	6	53	F	170	60	60	84	399
	7	53	F	176	79	67	82	389
	8	52	F	172	91	61	94	442
	9	62	F	165	54	56	78	411
	10	62	M	186	90	57	96	422
	11	50	M	176	62	55	100	442
	12	25	F	158	50	64	99	445
	13	37	F	164	57	55	58	423
	14	28	F	164	49	58	89	458
	15	49	F	174	72	66	90	423
	16	49	F	172	61	67	91	438
	17	56	F	168	68	67	93	440
	18	50	F	164	66	57	94	419
	19	40	F	168	64	71	90	433
	20	40	M	178	60	53	65	427
	21	21	M	180	80	63	79	418
	22	30	M	183	75	59	75	428
Pathological	RBBB	71	M	170	72	21	166	453
	LBBB Pat. A	77	F	162	66	27	132	486
	LBBB Pat. B	52	F	168	112	56	158	455
	LBBB Pat. C	51	F	179	79	53	150	474
	Symp. LQT2	49	F	180	84	63	82	500
	Symp. LQT3	38	M	190	87	53	96	486
	Asymp. LQT1	49	M	183	84	55	108	457
	Asymp. LQT3	36	F	161	53	59	72	438

F, female; LBBB, left bundle branch block; LQT, long-QT; LVEF, left ventricular ejection fraction; M, male; Pat., patient; QTcB, QT interval corrected according to Bazett’s formula; RBBB, right bundle branch block; SD, standard deviation.

LQTS patients are further divided into symptomatic (‘Symp.’; with history of ventricular tachyarrhythmias) and asymptomatic (‘Asymp.’; without such history).

### Study procedure

**Figure 1:**
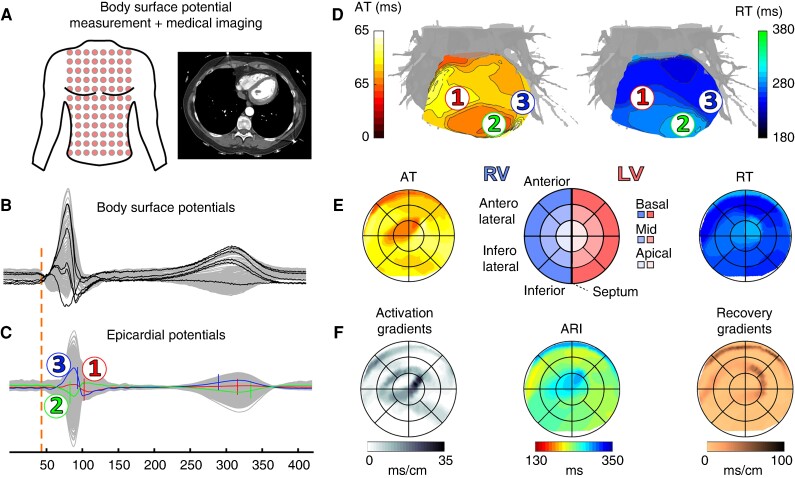
ECGI study procedure.^6^ (*A*) Body surface potential measurement and cardiothoracic imaging (CT or MRI) to visualize electrode positions and heart geometry. (*B*) Recorded body surface potentials before inverse reconstruction. (*C*) Epicardial reconstructed potentials for each virtual node on the ventricular surface. Numbers 1, 2 and 3 indicate examples of electrograms, sampled from indicated locations in panel *D*. (*D*) By determination of AT (steepest downslope of local QRS complex in *C*) and RT (steepest upslope of local T wave^9^ in *C*), activation and recovery isochronal maps were calculated. (*E*) Epicardial bullseye projection of isochronal AT and RT maps, with anatomical reference shown in the middle. (*F*) Bullseye projection of activation gradients, ARI, and recovery gradients, all derived from (*D*). ARI, activation–recovery interval; AT, activation time; LV, left ventricle; RT, recovery time; RV, right ventricle.

The study procedure and ECGI analysis are shown in *Figure 1*. In both MUMC + and ICL, ∼200 Ag–AgCl electrodes (ActiveTwo, BioSemi, The Netherlands) were attached to the participant’s torso to record body surface potentials at a sampling frequency of 2048Hz for 11 ± 7 min at rest (*Figure 1A*). At MUMC+, subjects then underwent a non-contrast low-dose thoracic CT scan to image the electrode positions and coronary CT angiography with intravenous administration of iodinated contrast medium to image the heart at end-diastole. Subjects at ICL received a 1.5-T MRI scan to image the heart geometry at end-diastole and the positions of MRI-safe markers that replaced the body surface electrodes at identical anatomical locations.

### Data processing and electrocardiographic imaging reconstruction

For each subject, 11 normally conducted sinus beats were selected from the body surface recording (*Figure 1B*) for inverse reconstruction. Out of these 11 beats, 10 were consecutive. One beat at a different point in time [separated by 268 (182–516) s, depending on recording duration] was randomly selected as a remote reference for comparison. For Subject 5, a premature ventricular complex (PVC) was also selected for reconstruction. Our previously validated ECGI methods (see Supplementary material online, *Figure S1* and^10,11^) were then used to reconstruct unipolar electrograms (UEGs) from the body surface potentials onto the ventricular epicardial surface (*Figure 1C*). For each epicardial UEG, the activation time (AT) and recovery time (RT) were automatically determined from the steepest downslope of the epicardial QRS complex and the steepest upslope of the epicardial T wave,^9^ respectively, using a spatiotemporal approach that considers the spatial flow of current.^12^ Isochronal AT and RT maps were visualized on the 3D heart surface (*Figure 1D*). For more details, see Supplementary material online, *Methods*.

### Standardized bullseye visualization

Our previously validated open-source algorithm (UNISYS^13^) was used for standardized epicardial bullseye visualization of the results (*Figure 1E*), allowing segmental analysis and comparison between different hearts. Results were compared between left ventricle (LV) and right ventricle (RV), circumferential segments (apex/centre/base), and radial segments (LV-lateral/anterior/RV-lateral/inferior).

### Outcome measures

After assigning AT and RT for each local UEG, metrics were determined from local AT and RT (*Figure 1F*). Activation–recovery interval (ARI), a surrogate measure of local action-potential duration,^9^ was calculated as the subtraction of local AT from local RT. Duration of activation (AT_DUR_) was quantified as the difference between the maximum and minimum local ATs of all nodes. Duration of recovery (RT_DUR_) was calculated similarly (difference between maximum and minimum local RTs of all nodes). Local spatial gradients of activation (ATG) and recovery (RTG) were defined as the largest local gradient (AT or RT difference divided by internode distance) in a 10-mm region. Activation–ARI relationships were investigated using linear regression with AT as the independent variable and ARI as the dependent variable (ARI = α ⋅ AT + β).

### Statistics

Unless otherwise noted, all outcome measures are averaged over 10 consecutive sinus beats to ensure consistency and minimize the influence of local outliers. Outcome measures are presented as average ± SD if normally distributed, and medians (first–third quartiles) are provided for non-normally distributed data (tested with the Shapiro–Wilk test). Unpaired two-tailed Student’s *t*-tests were used to compare normally distributed data, and the Mann–Whitney *U* test was used to compare non-normally distributed data. *P* ≤ 0.05 was considered significant. Outcome measures were compared between males and females and between subjects <50 and ≥50 years of age, resulting in two groups of approximately equal size. To compare the recovery of subjects <50 years with that of subjects ≥50 years, we selected those with similar RR intervals, as this influences recovery. Consequently, seven subjects were selected for each age group, with comparable RR intervals.

Intra- and inter-individual Pearson’s correlation coefficients (CCs) were calculated to compare ECGs, AT maps, and RT maps between and within subjects. For isochronal bullseye comparisons, we compared ATs and RTs at 20 standardized locations (the centre of each bullseye segment, see *Figure 1E*). For between-subject comparisons, the inter-individual CC of the fourth beat is reported, to ensure a regular heart rate when feasible. For within-subject comparisons, we report the average value of all consecutive beat-to-beat CCs (beats 1 vs. 2, 2 vs. 3, etc.) and the average CC of all consecutive beats vs. the beat remote in time (beat 1 vs. remote beat, beat 2 vs. remote beat, etc.).

## Results

### Characteristics of study subjects

*Table 1* summarizes the subject characteristics. The average age of normal subjects was 49 ± 14 years, and 6/22 were male. The average height of normal subjects was 179 ± 6 cm for males and 167 ± 6 cm for females. The median weight was 73 (62–80) kg for males and 65 (57–74) kg for females. Left ventricular ejection fraction, QRS duration, and QTc according to Bazett’s formula were within the normal range in all individuals. RR intervals were not significantly different between males and females (904 ± 184 ms vs. 905 ± 132 ms, respectively; *P* = 1.00) nor between age groups (828 ± 107 ms vs. 875 ± 81 ms, respectively; *P* = 0.38).

### Electrical activation and recovery of the left and right ventricles

**Figure 2:**
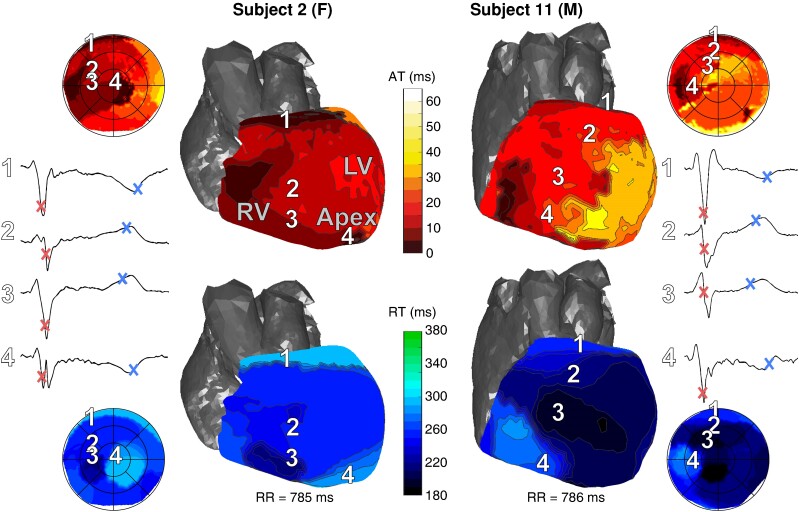
Illustrative examples of isochronal activation (top) and recovery (bottom) maps for a female (left) and male (right) subject. Numbers 1–4 indicate different locations on the heart and their corresponding electrograms. AT and RT are indicated by the markers on these electrograms. Bullseyes in the corners of the figure show the entire epicardium, whereas 3D views only show the anterior side. AT, activation time; F, female; LV, left ventricle; M, male; RT, recovery time; RV, right ventricle.

*Figure 2* shows activation and recovery maps for a male and female subject of a single normally conducted sinus beat, both showing earliest epicardial activation at the RV anterior base, with additional breakthroughs at other sites. Local UEGs at breakthrough sites showed an ‘rS’ morphology, indicative of an endo-to-epicardial activation pattern. Regional recovery patterns differed between these individuals.

**Figure 3:**
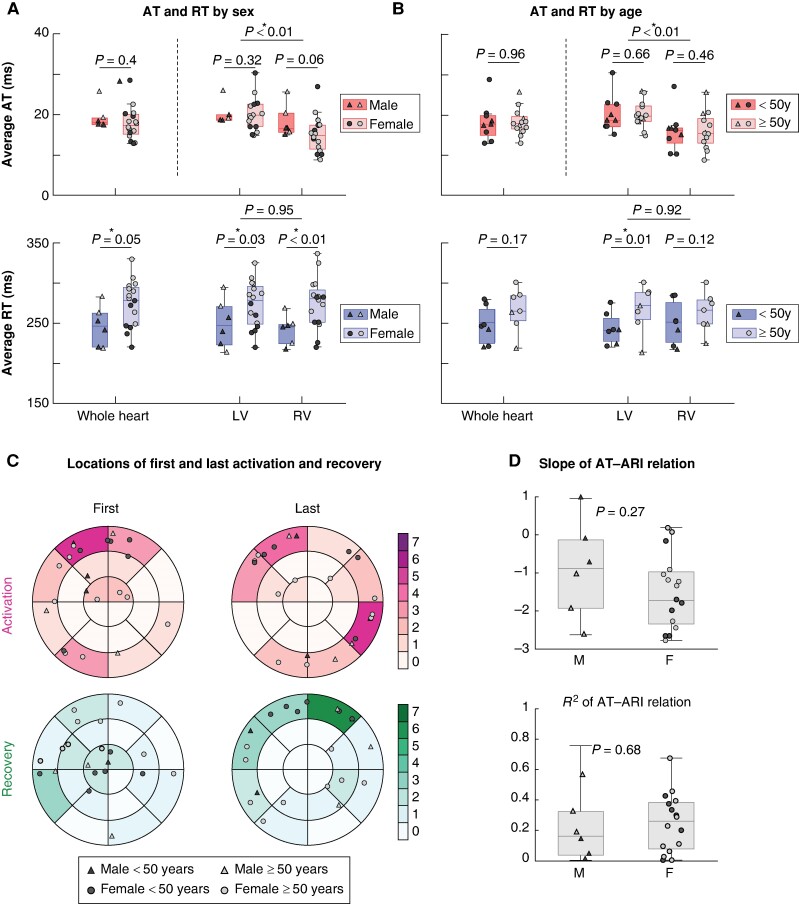
Overview of control results. (*A*) Average AT and RT for males and females. The top horizontal bar indicates *P*-value between LV and RV. Second-level horizontal bars indicate *P*-values between males and females. Asterisks above *P*-values (*) indicate significant differences. Triangles indicate males, circles indicate females, filled symbols indicate subjects <50 years, and open symbols indicate subjects ≥50 years. (*B*) Average AT and RT for subjects <50 and ≥50 years. (For RT, only seven subjects were included for each group, to ensure similar RR intervals.) (*C*) Locations of first and last areas of activation and recovery. (*D*) Slope and coefficient of determination of AT–ARI relation (see Results). ARI, activation–recovery interval; AT, activation time; F, female; LV, left ventricle; M, male; RT, recovery time; RV, right ventricle.

Activation and recovery characteristics for all 22 normal subjects averaged for 10 consecutive beats are shown in *Figure* *3A* and *B*. The average AT of the LV was 4 ms later than that of the RV (*P* < 0.01), while earliest AT was not significantly different between ventricles (1 ms earlier for RV, *P* = 0.49). Duration of activation was not significantly different between ventricles, see Supplementary material online, *Figure S2*. Three examples of activation maps, comparing the LV to the RV, are shown in Supplementary material online, *Figure S3*. The average AT did not differ significantly between sex (*P* = 0.4) or age groups (*P* = 0.96).

The average RT did not differ significantly between LV and RV (*P* = 0.95) but was shorter in males than in females (246 ± 25 vs. 274 ± 29 ms, *P* = 0.05). Recovery started significantly earlier in males (188 ± 25 vs. 222 ± 26; *P* = 0.01), but duration of recovery tended to be longer, with a borderline difference [116 (114–120) vs. 96 (85–111) ms; *P* = 0.06], see Supplementary material online, *Figure S2*. The average RT of LV was significantly shorter in subjects <50 years than in those ≥50 years (239 ± 20 vs. 267 ± 28 ms, respectively; *P* = 0.01), but not for the RV or whole heart.

Normalizing AT and AT_DUR_ for ventricular surface area did not result in significant differences between sex or age groups (*P* > 0.26 in both cases). Additionally, AT and AT_DUR_ did not correlate significantly with ventricular surface area (*P* > 0.25 in both cases). Further details on segmental analyses of AT and RT are provided in Supplementary material online, *Figure S4*. All values for ECGI-based metrics of controls are provided in the Supplementary Data file for future reference.

### Locations of first and last activation and recovery

*Figure 3C* shows the locations of the first and last activation and recovery of a normally conducted sinus beat for all normal subjects. First epicardial activation appeared at the anterior/anterolateral RV in 10/22 subjects. The location of the last activation occurred most frequently in the anterior/anterolateral RV (9/22) or inferior/inferolateral LV (8/22). Of the 10 subjects with earliest activation in the anterior/anterolateral RV, three individuals also had last activation in one of these segments (see also Supplementary material online, *Figure S5*). Earliest recovery typically occurred on the RV (14/22), whereas last recovery was more heterogeneous between subjects. In subjects <50 years, the locations of first and last recovery appeared to be less dispersed than in subjects ≥50 years.

### Activation-ARI relationship

The normal T wave on the 12-lead ECG is typically considered concordant with the QRS complex because of early activated tissue having a longer ARI and later activated tissue having a shorter ARI. Activation–ARI relationships (defined as ARI = α ⋅ AT + β) capture the relationship between local repolarization duration and the activation sequence. These had a negative slope on average, −1.30 ± 1.08 (−0.88 ± 1.3 for males vs. −1.46 ± 0.99 for females; *P* = 0.27), although slopes were heterogeneous between subjects (see *Figure 3D*). The average coefficient of determination (*R*^2^) was 0.24 ± 0.19 (0.21 ± 0.21 for males vs. −0.25 ± 0.19 for females; *P* = 0.68).

### Between-subject and within-subject variability

**Figure 4:**
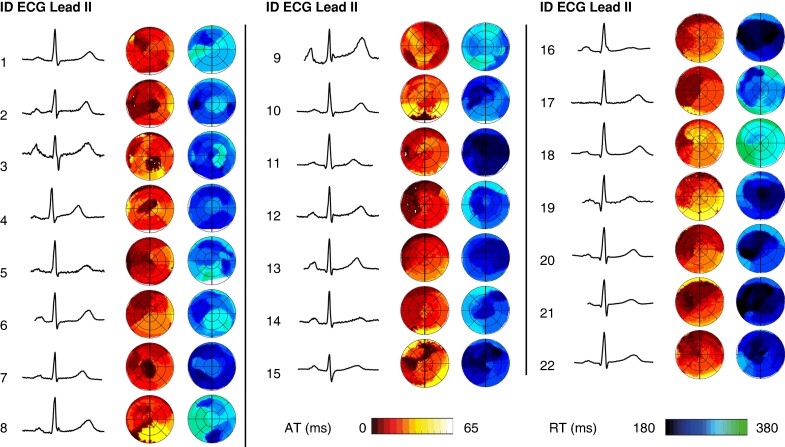
Lead II of the body surface ECG, together with their co-registered activation isochrones and recovery isochrones for all subjects.

**Figure 5:**
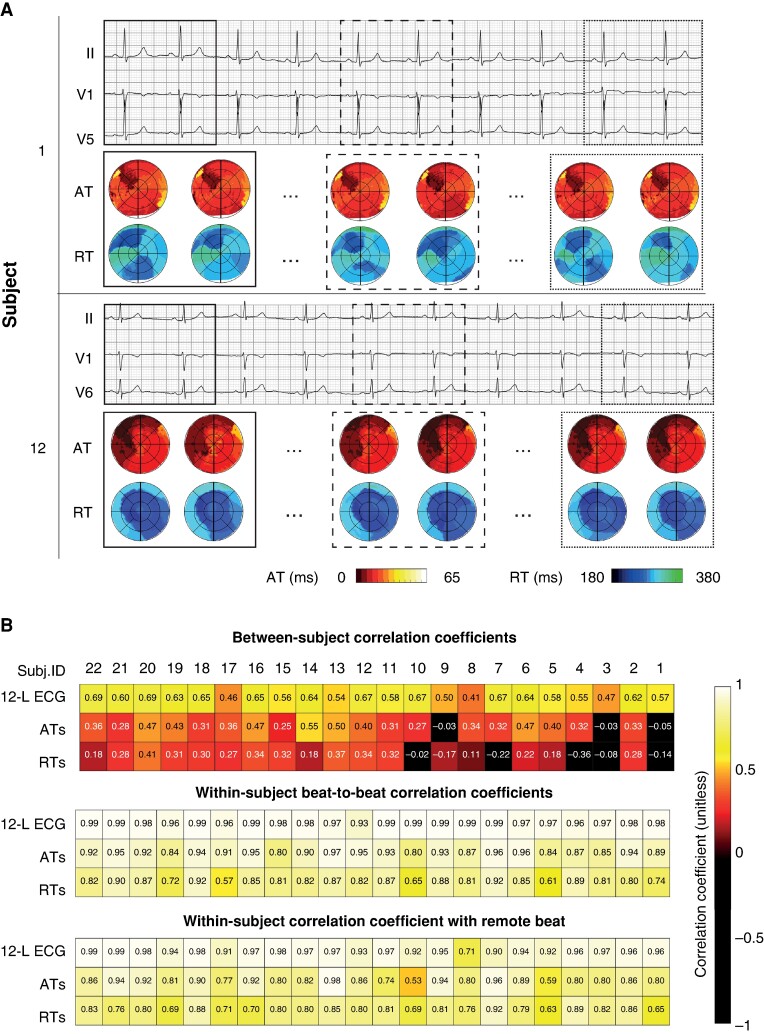
Within-subject and between-subject comparisons of ECG, activation maps, and recovery maps. (*A*) 3-Lead ECG and corresponding isochronal activation and recovery bullseyes of two selected subjects (Subjects 1 and 12). For each subject, activation and recovery of beats 1, 2, 5, 6, 9, and 10 are shown. Square boxes indicate which bullseyes correspond to which beats. (*B*) Correlation coefficients are used to compare 12-lead ECGs, activation maps, and recovery maps between and within subjects. For example, for between-subject comparisons, the median CC is noted for between-subject comparisons of Subject 1 to Subjects 2–22. For within-subject comparisons, the median value is noted for beat-to-beat comparisons of beat 1 vs. beat 2, beat 2 vs. beat 3, etc. ATs, activation times; CC, correlation coefficient; 12-L ECG, 12-lead ECG; RTs, recovery times.

*Figure 4* shows the comparison of ECG lead II and their co-registered activation and recovery bullseyes in all normal subjects. Whereas the ECG was normal, high-resolution AT and RT maps showed considerable variation among individuals. *Figure 5A* shows the ECGs of two subjects, together with their co-registered AT and RT maps. Within-subject AT and RT maps remained stable over 10 beats. 12-Lead ECGs, AT maps, and RT maps were quantitatively compared within and between subjects using CCs (see *Figure 5B*). The CC for 12-lead ECG comparisons of the fourth beat between subjects was 0.61 (0.46–0.72). Correlation coefficients for between-subject comparisons of AT and RT maps were 0.32 (0.07–0.50) and 0.20 (−0.12–0.42), respectively. On the other hand, CCs of within-subject beat-to-beat comparisons of ECGs, AT maps, and RT maps were 0.98 (0.97–0.99), 0.92 (0.87–0.94), and 0.74 (0.71–0.79), respectively, indicating a high within-subject stability of these outcome measures. When comparing the 10 consecutive beats within a subject to a beat remote in time, CCs were 0.95 (0.93–0.97) for comparisons of the 12-lead ECG, 0.82 (0.80–0.91) for AT maps, and 0.78 ± 0.08 for RT maps.

### Bundle branch block

**Figure 6:**
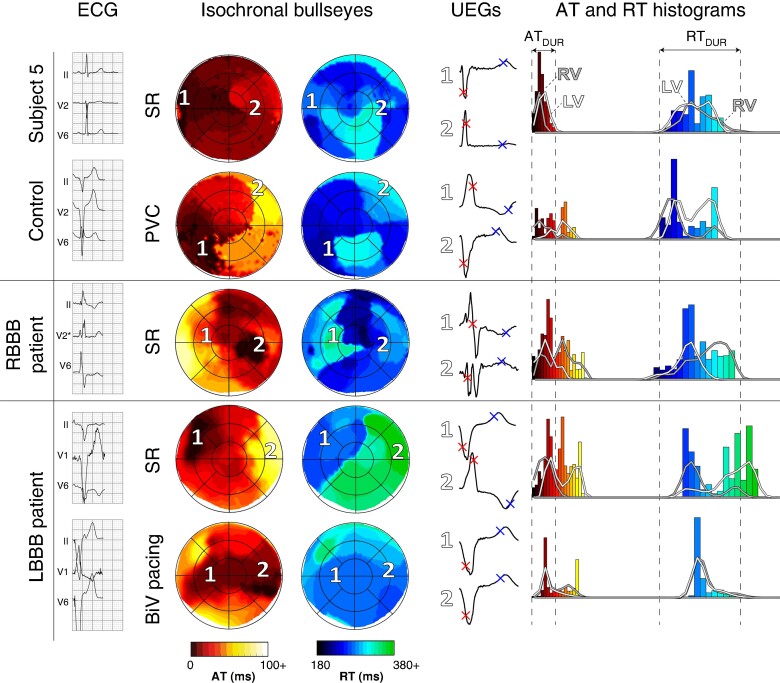
Comparison of activation and recovery patterns of a control subject in sinus rhythm (SR) and with a PVC (top), a subject with RBBB (middle), and a subject with LBBB during SR and with BiV pacing (bottom). Body surface ECGs, isochronal bullseyes with selected UEGs (indicated by numbers 1 and 2), and AT and RT histograms are shown from left to right. (Dys)synchrony in activation and recovery between LV and RV, as visible in isochronal bullseyes, is also reflected in the histograms. AT, activation time; AT_DUR,_ duration of activation; BiV, biventricular; LBBB, left bundle branch block; LV, left ventricle; PVC, premature ventricular complex; RBBB, right bundle branch block; RT, recovery time; RT_DUR_, duration of recovery; RV, right ventricle; SR, sinus rhythm; UEGs, unipolar electrograms.

*Figure 6* shows the activation and recovery patterns of a control subject, the RBBB subject, and an LBBB subject with and without biventricular (BiV) pacing. In the control subject, the RV activated first, AT_DUR_ was 45 ms, and RT_DUR_ was 135 ms. A PVC from the inferolateral RV caused non-synchronous activation, resulting in an AT_DUR_ of 83 ms and an RT_DUR_ of 95 ms.

In the RBBB subject, the non-synchronous activation caused the LV to activate first, resulting in a prolonged AT_DUR_ of 94 ms and RT_DUR_ of 204 ms. The LV activated and recovered before the RV.

In the LBBB subject, the non-synchronous activation pattern caused an AT_DUR_ of 87 ms and an RT_DUR_ of 126 ms. The RV activated and recovered before the LV. Short-term BiV of several beats resulted in comparable AT_DUR_ (91 ms) as during sinus rhythm, but partially resynchronized the LV and RV, resulting in early activated areas in both ventricles and more homogeneous recovery (RT_DUR_ 99 ms). Correlation coefficients of within-subject beat-to-beat comparisons of ECGs, AT maps, and RT maps of BBB patients were similar to those of controls (see Supplementary material online, *Figure S6*). Results of all BBB subjects are further compared quantitatively with controls in Supplementary material online, *Figure S7*. The agreement of our findings with literature data is shown in Supplementary material online, *Figures S8* (for activation-based measures) and *S9* (for recovery-based measures).

### Long-QT syndrome

**Figure 7:**
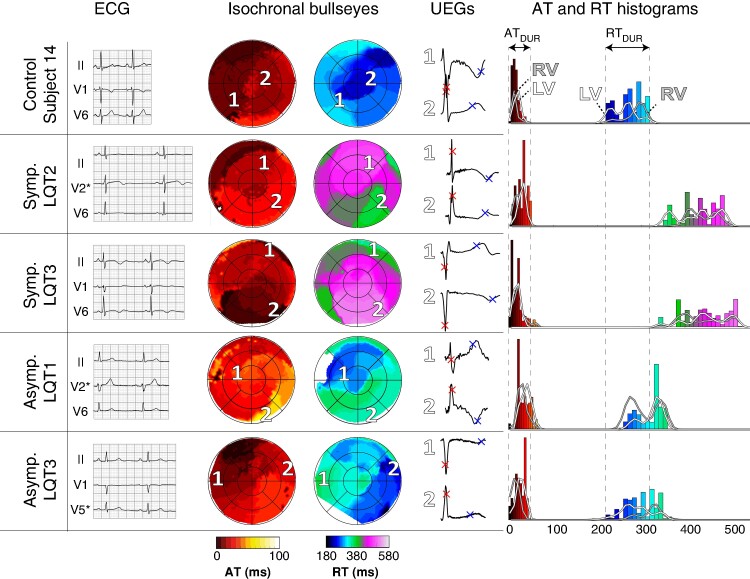
Comparison of activation and recovery patterns of five subjects in sinus rhythm: a control subject (top), two clinically overt LQTS patients with history of ventricular tachyarrhythmias (‘Symp. LQT2’ and ‘Symp. LQT3’) and two clinically occults without such history (‘Asymp. LQT1’ and ‘Asymp. LQT3’). Body surface ECGs, isochronal bullseyes with selected UEGs (indicated by numbers 1 and 2), and AT and RT histograms are shown from left to right. (Dys)synchrony in activation and recovery between LV and RV, as visible in isochronal bullseyes, is also reflected in the histograms. AT, activation time; AT_DUR,_ duration of activation; LQT, long-QT; LV, left ventricle; RT, recovery time; RT_DUR_, duration of recovery; RV, right ventricle; UEGs, unipolar electrograms.

*Figure 7* shows the comparison of activation and recovery patterns between a control subject and four subjects with congenital LQTS. The control subject, age- and sex-matched to the asymptomatic LQT3 patient (see *Table 1*), had an average AT of 13 ms and an average RT of 272 ms. Both symptomatic LQTS patients exhibited a prolonged QT interval and T-wave negativity, as seen on the ECG. This was also evident from ECGI, resulting in an average RT of 441 and 429 ms; the average AT was 20 and 25 ms, within the normal range. The asymptomatic LQTS patients had normal QT intervals on the ECG and a borderline average AT (28 and 34 ms). However, ECGI revealed an average RT of 307 and 315 ms, which was markedly longer than age- and sex-matched controls. Likewise, for BBB, CCs of within-subject beat-to-beat characteristics of LQTS patients were similar to those of controls (see Supplementary material online, *Figure S4*). Quantitative comparisons of pathological subjects to age- and sex-matched controls are provided in Supplementary material online, *Figure S7*. Agreement of our study results with literature data is shown in Supplementary material online, *Figures S8* and *S9*.

## Discussion

Using non-invasive ECGI, we have demonstrated that ventricular activation and recovery patterns can vary considerably among normal individuals, who all had unremarkable 12-lead ECGs. In each of these subjects, activation and recovery characteristics were relatively stable over the course of multiple beats to minutes. Recovery times were shorter in males than in females and at younger age (in the LV). We found only limited association between the sequence of activation and the duration of local recovery (ARI).

### Activation and recovery patterns vary greatly between control subjects, but are stable within individuals

Although all subjects had a normal 12-lead ECG, their underlying ventricular activation and recovery patterns were profoundly different. This appears consistent with the results of previous studies, although differences were not reported explicitly there.^4,5^ Individual characteristics such as conduction system anatomy, Purkinje network distribution, ion-channel expression, and autonomic innervation of the ventricular myocardium may underlie the variant activation and recovery patterns that we have revealed, and they may exaggerate under pathological conditions. The relative stability over multiple sinus beats in individual study subjects implicates that electrical perturbations during pathological conditions may be signalled by ECGI by revealing abnormal dynamics in the activation and recovery isochrones, potentially even before the emergence of ectopy, as is also hinted by recent other work.^14^ Likewise, dynamic alterations in the substrate, e.g. in relation to arrhythmic events during drug provocation testing, or heart rate changes in response to exercise, could be uncovered. This should be investigated in future studies.

### Activation characteristics

The epicardial locations of first (anterior to anterolateral RV) and last (RV anterior base or LV lateral base) activation that we found are consistent with findings from earlier studies.^5,7,8,15,16^ Interventricular variations in local wall thickness (including endocardial trabeculation, moderator band),^5^ myofibre orientation, and ion-channel expression (e.g. right-ventricular outflow tract)^4^ likely contributed to the sites of first and last activation and to the shorter average RV AT compared to the LV. Likewise, these factors could have caused inter-individual differences. Activation duration was similar to previous *ex vivo*^5^ and *in vivo*^15^ studies and 8 ms longer than in a non-invasive mapping study.^7^

No significant differences were found in average AT or AT_DUR_ between males and females. Most activation characteristics were similar between the age groups (<50 vs. ≥ 50 years), similar to a recent non-invasive mapping study.^17^

### Recovery characteristics

Epicardial locations of first and last recovery were more variable between subjects than those of activation. First recovery mostly occurred in the RV anterobasal segments, whereas last recovery mostly occurred in RV/LV anterobasal segments. This is in agreement with findings in three explanted human hearts.^4^ Epicardial recovery duration was similar to previous experimental work,^4^ and ARIs [246 (219–264) ms] were similar to an earlier non-invasive mapping study.^7^ There was a higher inter-individual variation of recovery than that of activation. Within-subject correlations of beat-to-beat RT maps were also lower than those of AT. Recovery time may thus be more variable between beats, but we cannot rule out that the larger variation derives, at least partly, from computational difficulties of determining RT.^18^

No significant differences were found in the average RT or RT_DUR_ between LV and RV. This contrasts with the earlier study of three explanted hearts^4^ but is in line with an earlier non-invasive mapping study.^7^

Males typically had a shorter average RT than females, as male electrical recovery started earlier and was not compensated by a longer recovery duration [a higher value of (last RT–first RT)]. Similarly, we found that the average RT was shorter in younger (<50 years) than in older subjects (≥50 years), as shown before.^17^

### Activation-ARI relationship

Previous human studies investigating the activation–ARI relationship were performed in diseased patients^1,2,19^ or *ex vivo* hearts,^3^ but *in vivo* results in normal subjects under physiological conditions were lacking. We found no strong relationship between the epicardial activation timing and ARI. The AT–ARI relationships over the epicardium (LV *plus* RV) were negative but with weak correlation [slope −1.30 ± 1.14 and *R*^2^ 0.23 (0.07–0.35)]. This indicates that the local ARI does not only depend on activation, but probably involves many other properties such as electrotonic coupling, local ion-channel expression, autonomic innervation, and hormonal influences. These results contrast with previous *in vivo* studies,^1,2^ but align with an *ex vivo* study,^3^ which may be explained by differences in study cohorts, mapping techniques, settings in which the recordings were performed, and the extensiveness of mapping.

### Pathological conditions

Activation (and consequently recovery) patterns in subjects with BBB were different from controls. LBBB and RBBB resulted in less synchronized and prolonged activation, in agreement with previous ECGI findings.^16,20–22^ Subsequent BiV pacing for LBBB resulted in an increased LV–RV synchrony of activation and recovery in the LBBB subject. While increased activation synchrony is thought to be supportive of mechanical function, increased recovery synchrony is considered antiarrhythmic.^11^ Electrical recovery was significantly prolonged in the subject with clinically overt LQTS (Patient A) in both the 12-lead ECG and ECGI, in agreement with previous ECGI findings.^23^ Moreover, the RT histogram was more widespread, indicating dispersion. Whereas the 12-lead ECG of the subject with clinically occult LQTS (Patient B) was normal, ECGI revealed prolonged recovery compared to age- and sex-matched controls, which would not have been recognized otherwise.

Consistent with other literature reports, our results illustrate the value of ECGI in non-invasively detecting and localizing AT and RT abnormalities with high detail, thus enabling a better characterization of proarrhythmic substrates, the dynamics of arrhythmia, and the effects of therapy.

### Study limitations

We used our previously validated implementation of ECGI^10,24^ to non-invasively assess epicardial electrophysiology, which did not allow to directly study endocardial activation and recovery. In some aspects, such as the determination of lines of conduction block, non-invasive mapping is less accurate than invasive mapping.^6,10,25^ However, invasive mapping is an expensive, complication-sensitive, and time-consuming method, and it would be unethical to perform this in non-diseased subjects. ECGI (with ∼1-cm resolution) does not reach the level of detail of invasive mapping (with presumed submillimeter resolution), but still enables to vastly enhance our understanding beyond the 12-lead ECG (without challenging the undisputed clinical value of the ECG). For a detailed overview of ECGI’s accuracy and a dissemination of its limitations, we refer to^6^

Our standardized bullseye visualization (UNISYS^13^) enabled standardized comparisons between epicardial segments within and between subjects, but thereby omitted the location of 3D anatomic structures that may vary between subjects, such as the outflow tracts and coronary arteries. Although we distinguished between males and females and young and old subjects, we did not account for other potential confounding factors, such as the menstrual cycle phase, autonomic tone, or time of recording (all of which could have influenced activation and recovery duration, RR interval, ion-channel expression, and/or arrhythmia susceptibility). Finally, our cohort was predominantly female. Although women have been historically underrepresented in cardiovascular research, we recognize the possibility that our results could be biased by the relative weight of female characteristics.

## Conclusions

Despite similar 12-lead ECGs, electrical activation and recovery vary significantly between normal individuals, while remaining stable within the same individual over multiple consecutive beats and over minutes of time. Our study sets a new standard for understanding ventricular electrophysiology in normal males and females of different ages. Individual ECGI characteristics may serve as a basis to better understand and monitor the development of electropathologies and arrhythmia.

## Supplementary Material

euae172_Supplementary_Data

## Data Availability

All relevant data are within the manuscript and its Supporting Information files.
