# Supplementary material for: Variant patterns of electrical activation and recovery in normal human hearts revealed by noninvasive electrocardiographic imaging
Source: Europace. 2024 Jul 5;26(7):euae172. doi: 10.1093/europace/euae172 (PMC11226755; doi:10.1093/europace/euae172)
Supplement: euae172_Supplementary_Data [file euae172_supplementary_data.zip › 20240617_Supplementary_Material.docx]

# Supplementary Material

**Variant patterns of electrical activation and recovery in normal human hearts revealed by noninvasive electrocardiographic imaging**

Job Stoks, Kiran Haresh Kumar Patel, Bianca van Rees, Uyen Chau Nguyen, Casper Mihl, Peter M Deissler, Rachel MA ter Bekke, Ralf Peeters, Johan Vijgen, Paul Dendale, Fu Siong Ng, Matthijs JM Cluitmans, Paul GA Volders.

**Contents**

[Supplementary Methods 2](#_Toc152618979)

[Anatomical images 2](#_Toc152618980)

[Body-surface potential maps 2](#_Toc152618981)

[Inverse reconstruction 2](#_Toc152618982)

[Supplementary Figures 5](#_Toc152618983)

[References 9](#_Toc152618984)

## Supplementary Methods

For a stable and accurate inverse solution, ECGI requires several key components: validation, processing of anatomical images, preprocessing of body-surface potential maps, and the inverse reconstruction. The inverse reconstruction can be further subdivided into the choice of ECGI formulation, the transfer matrix, regularization, and activation/recovery time determination and postprocessing. We provide a concise overview of methods here, for each of these components.

### Validation

The ECGI methodology and data processing of the current study were similar to our previous validation studies ^1,2^. A summary of our in-vivo validation study can be found in Supplementary Figure 1.

### Anatomical images

Electrode positions and epicardial geometry were manually segmented from the anatomical images, with the Seg3D software ^3^. For each individual, a torso geometry consisting of the centroids of electrode positions and a ~2000-nodal epicardial geometry were then digitized. The latter was generated by downsampling the exported segmentation and performing Laplacian smoothing in FEBio Preview ^4^.

### Body-surface potential maps

Noisy body-surface signals were semi-automatically selected and removed, resulting in a remaining 173±30 electrode signals. Baseline drift and 50 Hz noise were removed from the remaining body-surface potentials. Individual beats were manually selected, and their QRS complexes and T-waves were manually annotated. T-waves were filtered with a second-order 40 Hz low pass Butterworth filter ^5^.

### Inverse reconstruction

#### ECGI formulation

Previously validated ECGI methods ^1,2^ were used to reconstruct unipolar electrograms (UEG) on the ventricular epicardial surface for selected beats (Figure 1C). Inverse reconstruction of epicardial potentials was performed with the potential-based formulation of ECGI, which relies on the numerical relation between electrical potentials at the heart and body surface ^6^.

#### Transfer matrix

For each subject, we established the electrostatic relationship between the torso electrodes and epicardial ventricular nodes using a transfer matrix ^7^ through publicly available methods^8^. These methods assume homogeneous conductivity throughout the torso, resulting in comparable ECGI accuracy to an inhomogeneous torso model^9^, with improved ease of use. The transfer matrix is created with both the torso and heart considered non-moving. In-vivo and computational studies have shown that the effects of contractile and respiratory motion on common outcome measures in ECGI are small in physiological scenarios ^10,11^.

#### Regularization

Subsequently, we applied zeroth-order Tikhonov regularization ^12^ to constrain the solution to the inverse problem. For each cluster of either one or ten beats, a single Tikhonov parameter (λ) was used for all time instants to improve the temporal consistency of the reconstruction, which is important for UEG quality. This λ was chosen as the median of all values obtained with the L-curve method ^13^ for the different time instants during the QRS complexes and T-waves.

#### AT and RT determination

For each epicardial UEG, the activation time (AT) and recovery time (RT) were automatically determined from the steepest downslope of the epicardial QRS complex and the steepest upslope of the epicardial T-wave ^14^, respectively, using a spatiotemporal approach. This approach considers the spatial flow of current and is more accurate than a temporal-only approach ^15^. ATs and RTs were determined relative to the average moment of steepest QRS downslope of the first 25 activated nodes as a common time reference. Isochronal AT and RT maps were visualized on the 3D epicardial surface (Figure 1D).

#### RT post-processing

To ensure that outliers in RT annotation caused by noise were excluded, 1) local RTs within the first or last 10 ms of the STT-segment were blanked, 2) local RTs outside the 2^nd^-to-98^th^ percentile of all RTs were blanked, and 3) for each electrogram, RT was blanked if it fell outside the area of the largest positive derivative of the local T-wave, an adapted version of the previously published “confidence score” ^16^. A 15-mm spatial median filter was applied to remove noisy outliers and interpolate regions that were blanked ^2^. RTs were then manually verified. Finally, any areas of RTs that were manually determined to be erroneous (due to noisy UEG T-waves) were blanked and spatially interpolated. The percentage of blanked nodes by each of these steps is shown in Supplementary Table 1.

## Supplementary Figures

| 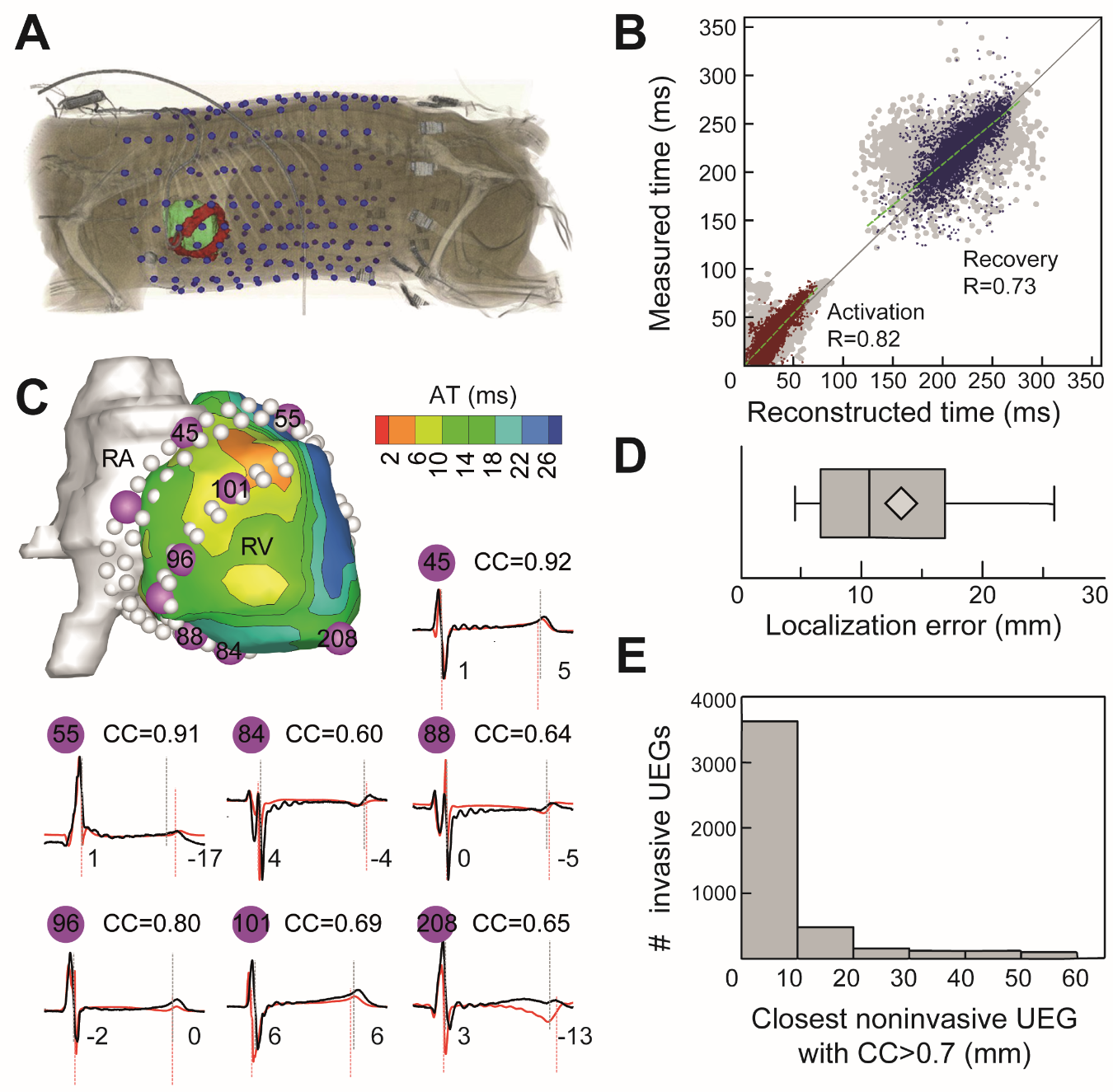 | |  |
| --- | --- | --- |
| **Supplementary Figure 1:** In-vivo validation of ECGI^1^. **A:** Experimental setup as applied in normal anesthetized dogs, illustrating body-surface electrodes (blue), epicardial surface (green), and epicardial contact electrodes (red). **B:** Scatter plot of invasively-measured vs. ECGI-reconstructed activation (R=0.82) and recovery times (R=0.73). **C:** Epicardial surface, with purple spheres indicating the position of epicardial electrodes. For the numbered purple spheres, recorded (red) and ECGI-reconstructed (black) UEGs are depicted below. Grey and red dashed lines indicate recorded and reconstructed activation/recovery times, respectively. The mismatch between both is given in milliseconds at the right for each pair. **D:** Box plots for localization mismatch for 80 paced beats in 4 dogs. Localization mismatch is deﬁned as the distance between the known pacing location and the location of earliest activation from non-invasively reconstructed UEGs. **E**: Histogram showing the spatial accuracy of ECGI, defined as the distance of each invasive UEG to the nearest noninvasive UEG with a good-enough correlation (CC>0.7). | |  |
| 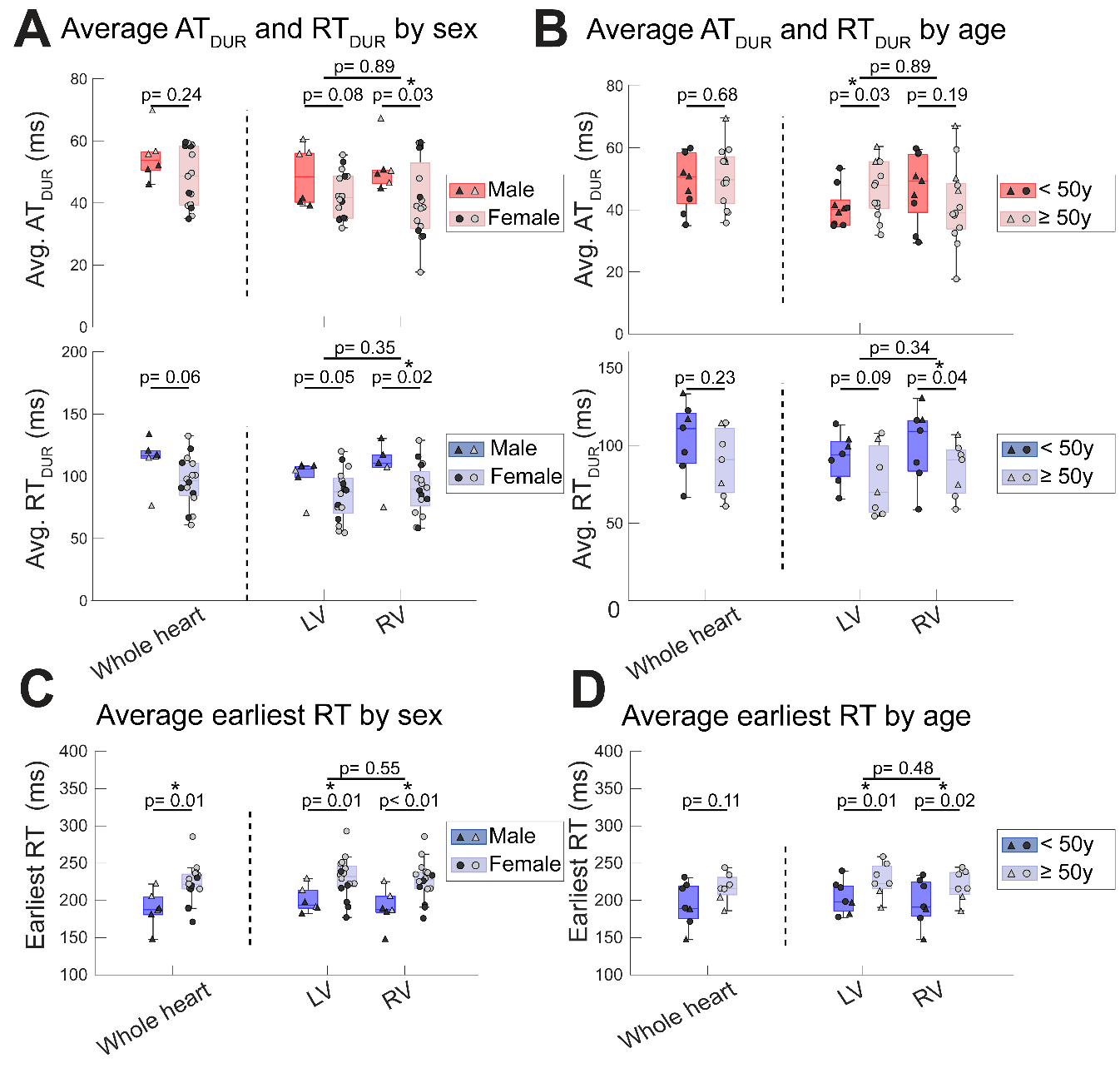 | | |
| **Supplementary Figure 2**: Average activation and recovery duration (AT_DUR_ and RT_DUR_, respectively) by sex and age (<50 y vs. ≥50 y). The top horizontal bars indicate p-value between segments. Second-level horizontal bars indicate p-values between males and females. For RT comparisons in B and D, only 7 subjects were included for each group, to ensure similar RR intervals. | | |
| 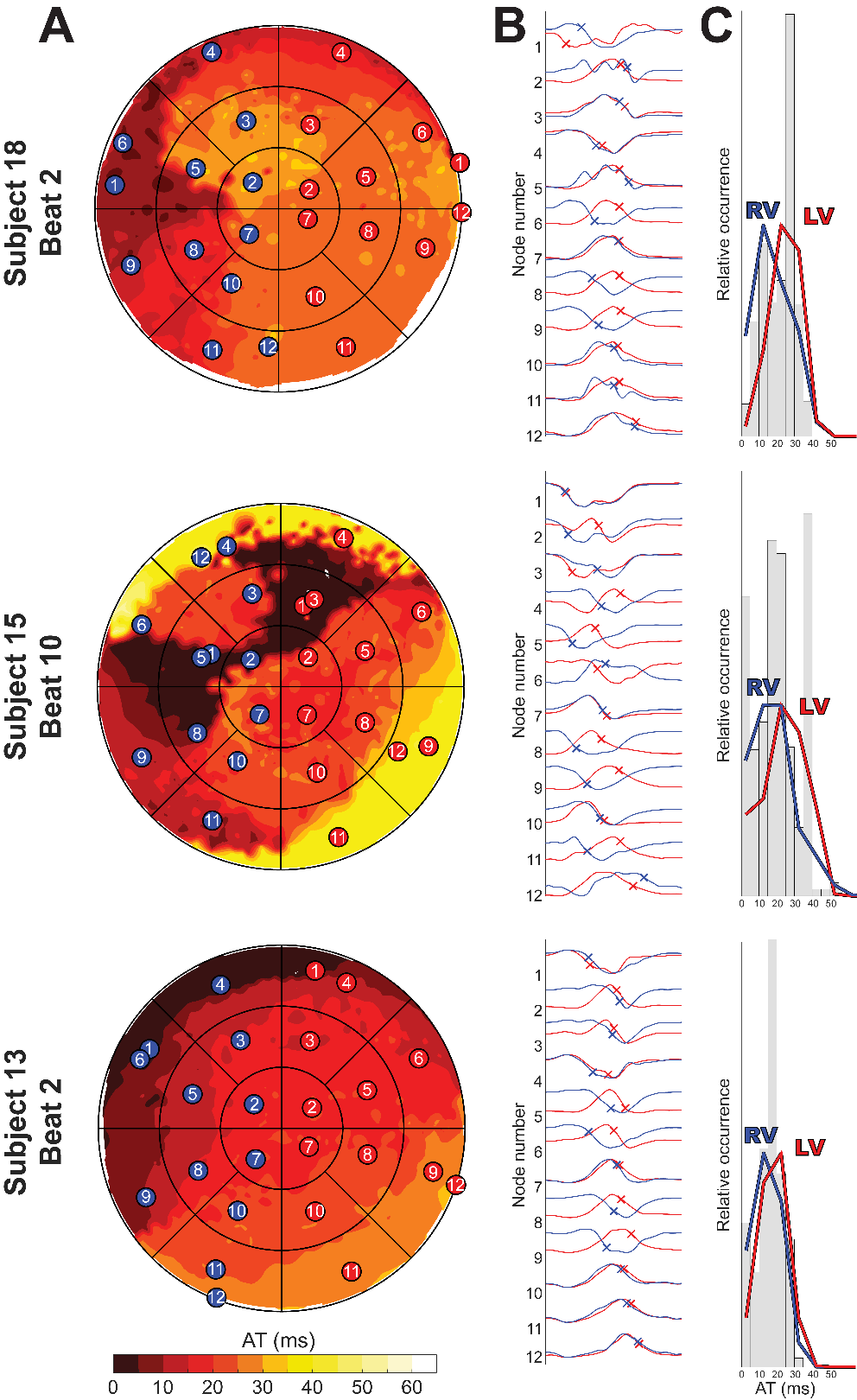 | |  |
| **Supplementary Figure 3**: Three example of activation maps. A: AT map with 12 markers indicating positions of sampled unipolar electrograms (UEGs) shown in panel B. Markers on the LV are shown in red, markers on the RV in blue. Markers 1 and 12 indicate the locations of first and last activation for each ventricle, respectively. B: UEGs of local QRS-complexes corresponding to markers in panel A. Blue UEGs correspond to the RV, red UEGs to the LV. X’s indicate annotations of local ATs. C: Histogram of ATs. The red histogram indicates ATs of both ventricles combined. The superimposed blue curve indicates ATs of the RV, the red curve indicates ATs of the LV. Even though earliest AT and AT_DUR_ were typically similar for both ventricles, average AT was earlier for the RV. | |  |
| 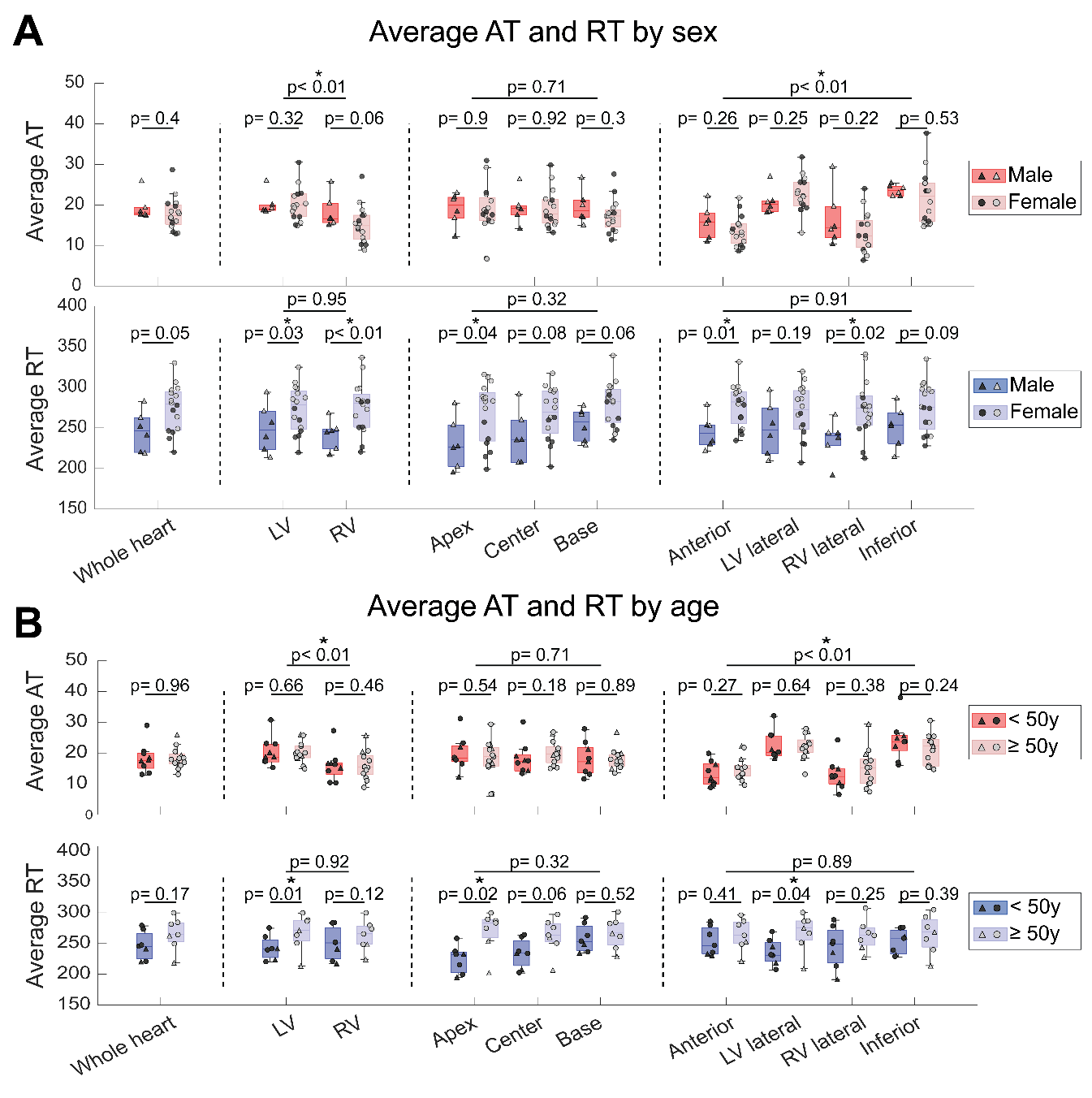 |  |  |
| **Supplementary Figure 4**: Average AT and RT by sex (A) and age (<50 y vs. ≥50 y, B), for all segments. The top horizontal bars indicate p-values between segments. Second-level horizontal bars indicate p-values between males and females. For RT comparisons in B, only 7 subjects were included for each group, to ensure similar RR intervals. |  |  |

| 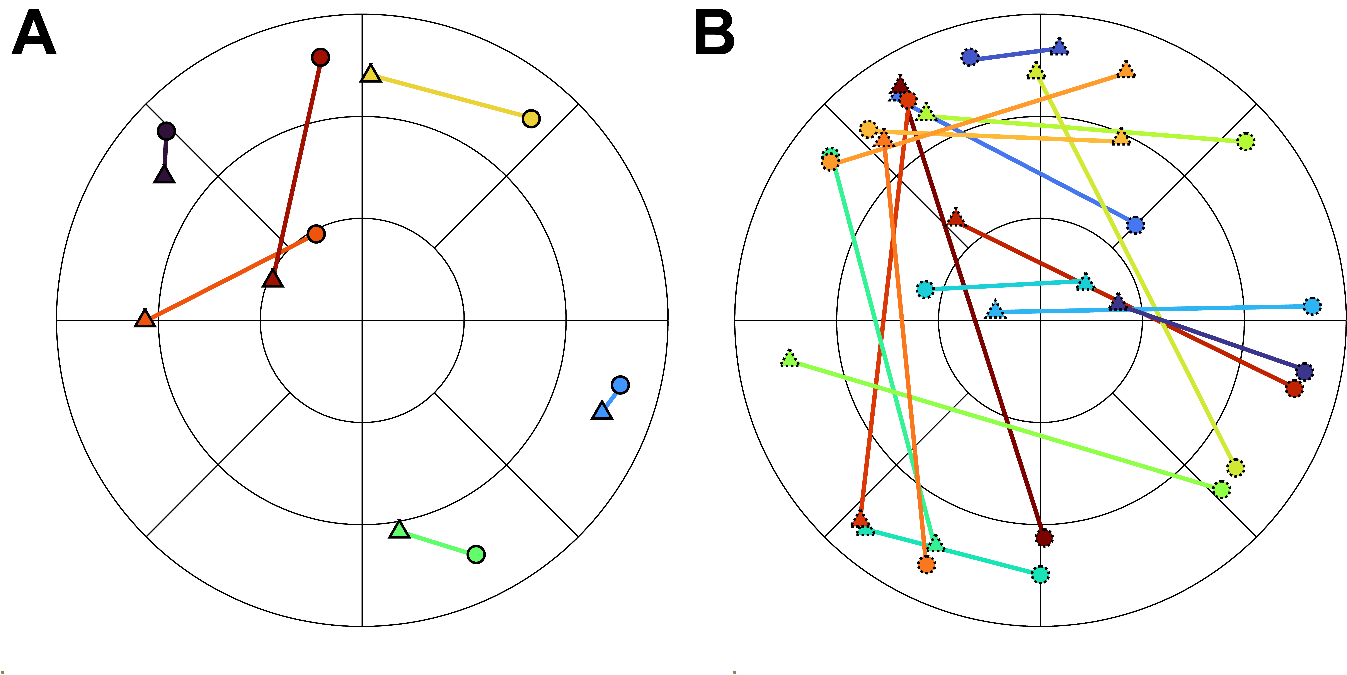 |
| --- |
| **Supplementary Figure 5:** Epicardial bullseye plots indicating locations of first and last activations for all subjects. Triangles indicate locations of first activation, while circles indicate locations of last activation. A: Solid symbols indicate the 6 individuals where the locations of first and last activation occurred in the same quartile. B: Dashed symbols indicate the 16 cases where these occurred in different quartiles. |

| 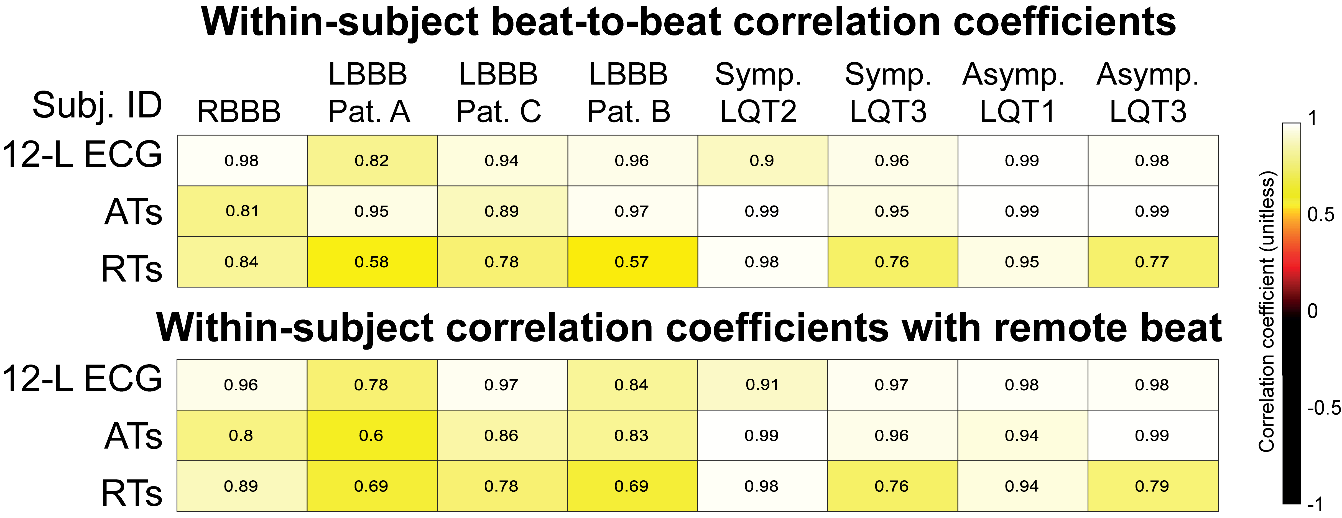 |
| --- |
| **Supplementary Figure 6:** Within-subject comparisons of ECG, activation maps and recovery maps for pathological subjects (see Figure 5 for values of controls). CCs are used to compare 12-lead ECGs, activation maps, and recovery maps within subjects. The median value of CCs is noted for beat-to-beat comparisons of beat 1 vs. beat 2, beat 2 vs. beat 3, etc. |
| 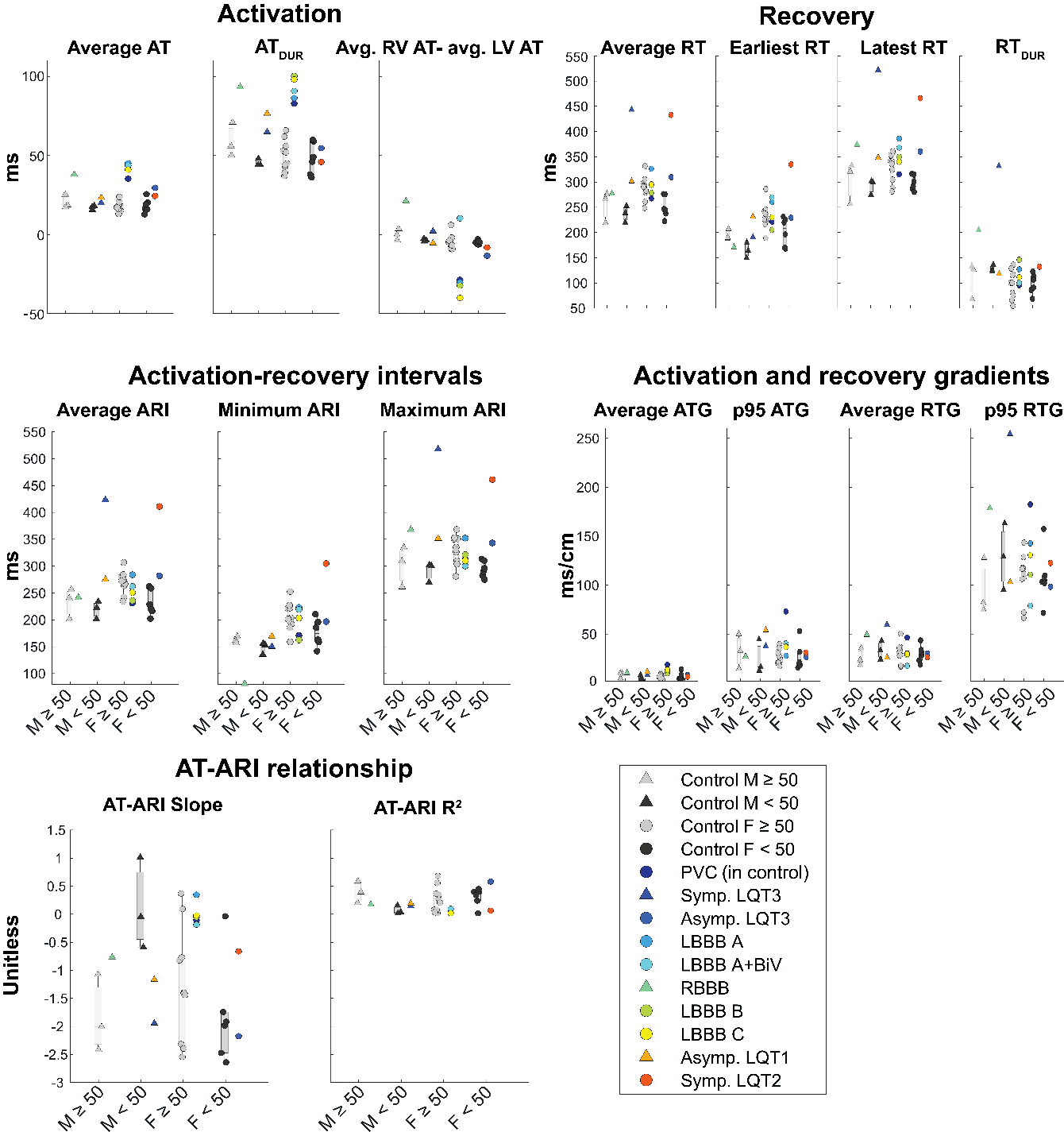 |
| **Supplementary Figure 7:** comparison of important outcome measures between healthy and pathological individuals, stratified by age and sex. P95: 95^th^ percentile. |

| 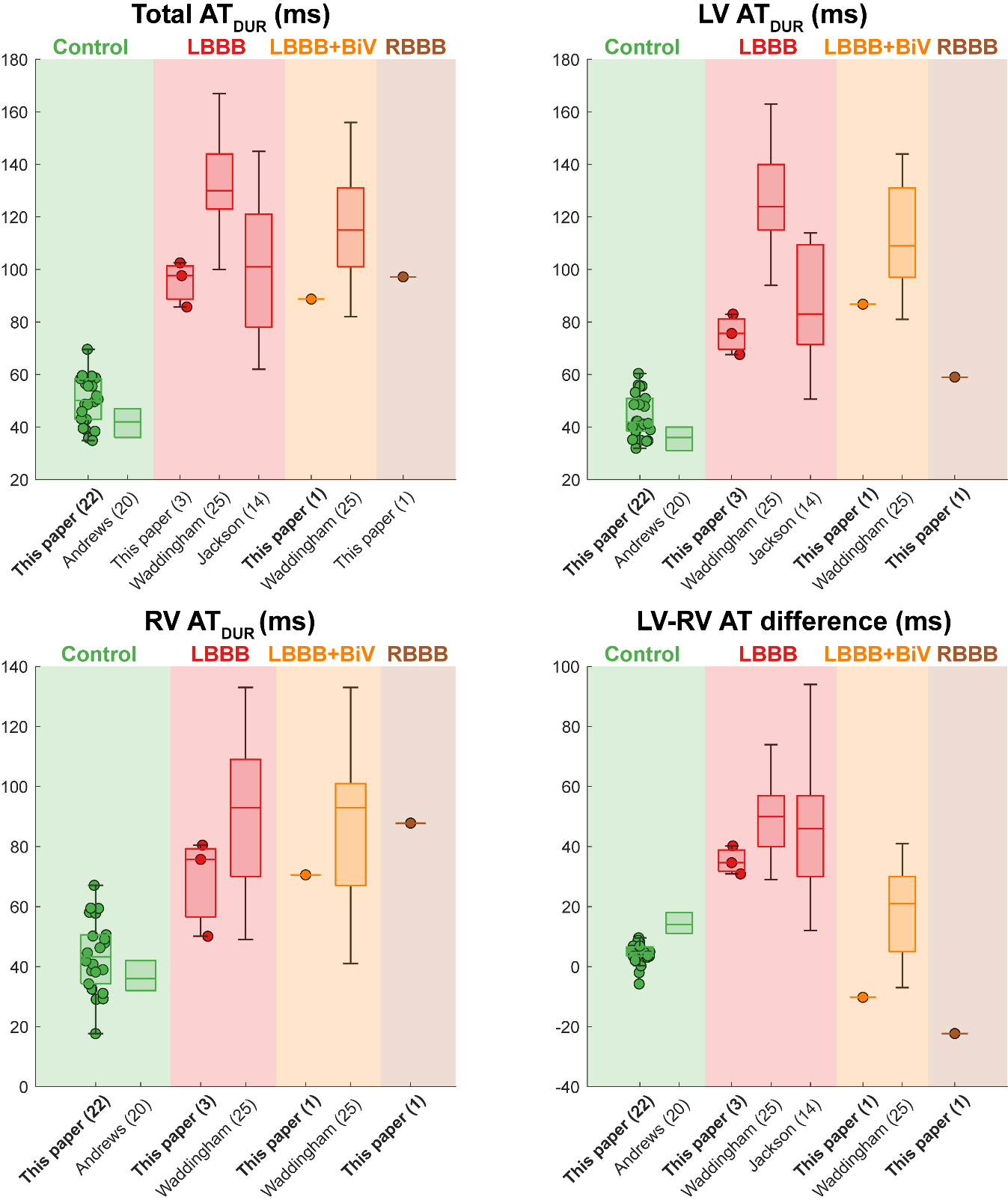 |
| --- |
| **Supplementary Figure 8:** Boxplots showing agreement of our activation-based study results with literature data^18–20^. Each graph indicates one outcome measure. Colors indicate conditions: control (green), LBBB (red), LBBB+BiV (orange), RBBB (brown). Within one color, different studies are shown on the x-coordinates, with the number of subjects shown in round brackets (*n*). Small circles indicate individual datapoints. |

| 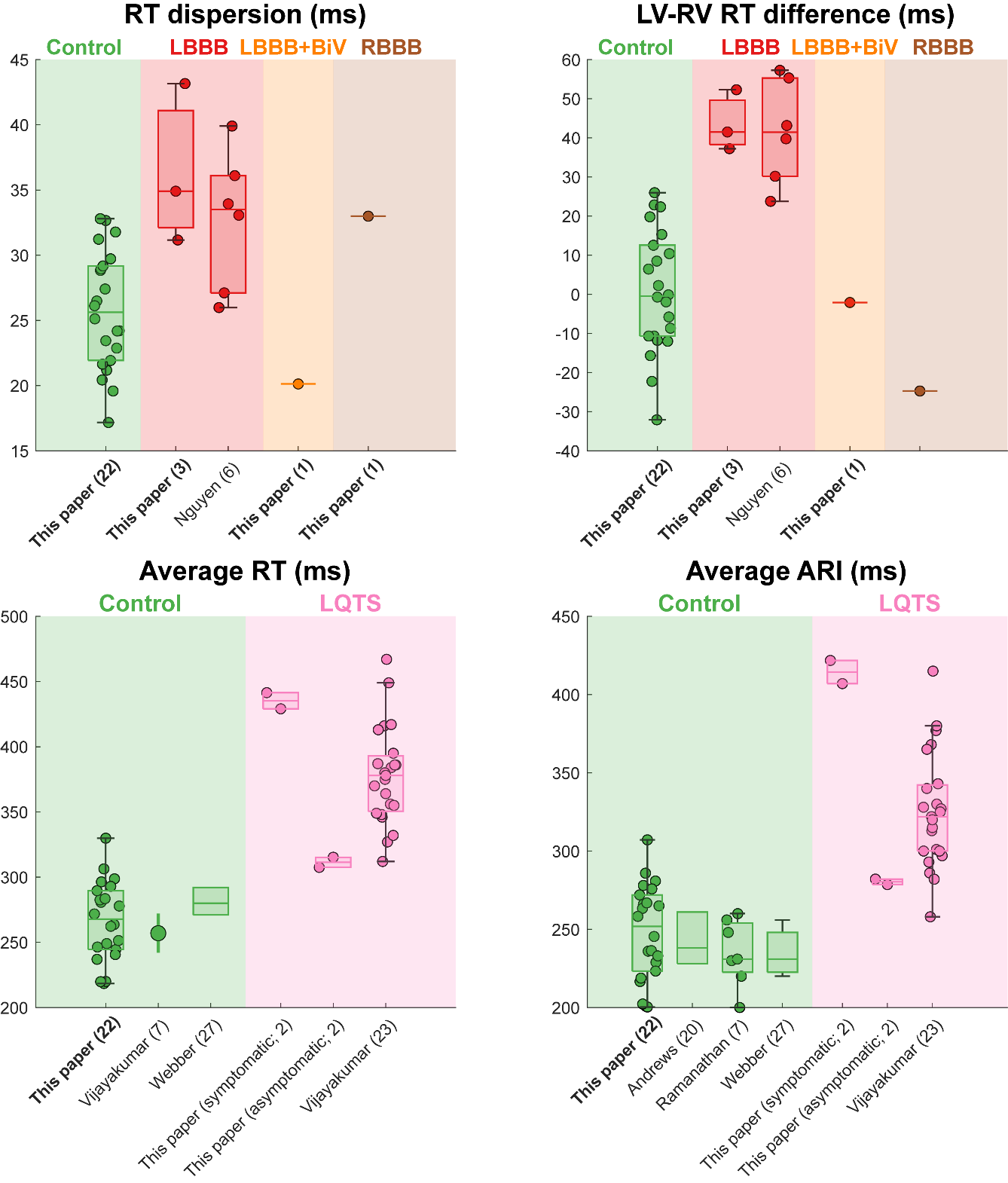 |
| --- |
| **Supplementary Figure 9:** Boxplots showing agreement of our recovery-based study results with literature data^18,21–24^. Each graph indicates one outcome measure. Colors indicate conditions: control (green), LBBB (red), LBBB+BiV (orange), RBBB (brown), LQTS (pink). Within one color, different studies are shown on the x-coordinates, with the number of subjects shown in round brackets (*n*). Small circles indicate individual datapoints, while large circles with vertical lines indicate average ± SD. |

## Supplementary Tables

**Supplementary Table 1**: Results of RT post-processing. We post-processed RTs semi-automatically in 4 steps (each shown in a separate column), to ensure that outliers caused by noise were excluded. Median percentage of blanked RTs (normalized for ~2000 nodes (= reconstructed UEGs) on the epicardial geometry) are shown for each of these steps.

| Earliest/latest 10ms | Outside the 2^nd^-to-98^th^ percentile of all RTs | Outside area of largest local T-wave positive derivative | Manual blanking |
| --- | --- | --- | --- |
| 0.6 (0.3-1.2) % | 2.2 (1.7-2.4) % | 3.6 (2.3-9.5) % | 2.6 (0-6.8) % |

# References

1. Cluitmans MJM, Bonizzi P, Karel JMH, Das M, Kietselaer BLJH, de Jong MMJ, et al. In Vivo Validation of Electrocardiographic Imaging. JACC Clin Electrophysiol. 2017;3(3):232–42.

2. Cluitmans MJM, Bear LR, Nguyên UC, van Rees B, Stoks J, ter Bekke RMA, et al. Noninvasive detection of spatiotemporal activation-repolarization interactions that prime idiopathic ventricular fibrillation. Sci Transl Med. 2021 Nov 17;13(620):1–11.

3. CIBC. Seg3D: Volumetric Image Segmentation and Visualization. Scientific Computing and Imaging Institute (SCI) [Internet]. 2016. Available from: http://www.seg3d.org

4. Maas SA, Ellis BJ, Ateshian GA, Weiss JA. FEBio: finite elements for biomechanics. J Biomech Eng. 2012 Jan;134(1):011005.

5. Ricciardi D, Cavallari I, Creta A, Di Giovanni G, Calabrese V, Di Belardino N, et al. Impact of the high-frequency cutoff of bandpass filtering on ECG quality and clinical interpretation: A comparison between 40 Hz and 150 Hz cutoff in a surgical preoperative adult outpatient population. J Electrocardiol. 2016;49(5):691–5.

6. Cluitmans MJM, Peeters RLM, Westra RL, Volders PGA. Noninvasive reconstruction of cardiac electrical activity: Update on current methods, applications and challenges. Neth Heart J. 2015;23(6):301–11.

7. Barr RC, Ramsey M, Spach MS. Relating Epicardial to Body Surface Potential Distributions by Means of Transfer Coefficients Based on Geometry Measurements. IEEE Trans Biomed Eng. 1977;BME-24(1):1–11.

8. Burton BM, Tate JD, Erem B, Swenson DJ, Wang DF, Steffen M, et al. A toolkit for forward/inverse problems in electrocardiography within the SCIRun problem solving environment. Annu Int Conf IEEE Eng Med Biol Soc IEEE Eng Med Biol Soc Annu Int Conf. 2011;2011:267–70.

9. Ramanathan C, Rudy Y. Electrocardiographic imaging: II. Effect of torso inhomogeneities on noninvasive reconstruction of epicardial potentials, electrograms, and isochrones. J Cardiovasc Electrophysiol. 2001 Feb;12(2):241–52.

10. J. Stoks, M. J. M. Cluitmans, R. Peeters, P. G. A. Volders. The Influence of Using a Static Diastolic Geometry in ECG Imaging. In: 2019 Computing in Cardiology (CinC). 2019. p. Page 1-Page 4.

11. Bergquist JA, Coll-Font J, Zenger B, Rupp LC, Good WW, Brooks DH, et al. Reconstruction of cardiac position using body surface potentials. Comput Biol Med. 2022;142(November 2021):105174.

12. Tikhonov AN, Goncharsky AV, Stepanov VV, Yagola AG. Numerical Methods for the Solution of Ill-Posed Problems [Internet]. Dordrecht: Springer Netherlands; 1995. Available from: http://link.springer.com/10.1007/978-94-015-8480-7

13. Hansen PC, O’Leary DP. The Use of the L-Curve in the Regularization of Discrete Ill-Posed Problems. SIAM J Sci Comput. 1993;14(6):1487–503.

14. Stoks J, Bear LR, Vijgen J, Dendale P, Peeters R, Volders PGA, et al. Understanding repolarization in the intracardiac unipolar electrogram: A long-lasting controversy revisited. Front Physiol. 2023 Apr 7;14.

15. Cluitmans M, Coll-Font J, Erem B, Bear L, Nguyên UC, ter Bekke R, et al. Spatiotemporal approximation of cardiac activation and recovery isochrones. J Electrocardiol. 2022;71:1–9.

16. Duchateau J, Potse M, Dubois R. Spatially Coherent Activation Maps for Electrocardiographic Imaging. IEEE Trans Biomed Eng. 2017;64(5):1149–56.

17. Bear LR, Cluitmans M, Abell E, Rogier J. Electrocardiographic Imaging of Repolarization Abnormalities. J Am Heart Assoc. 2021;10(020153).

18. Andrews C, Cupps BP, Pasque MK, Rudy Y. Electromechanics of the Normal Human Heart in Situ. Circ Arrhythm Electrophysiol. 2019;12(11):1–3.

19. Waddingham PH, Mangual JO, Orini M, Badie N, Muthumala A, Sporton S, et al. Electrocardiographic imaging demonstrates electrical synchrony improvement by dynamic atrioventricular delays in patients with left bundle branch block and preserved atrioventricular conduction. Eur Eur Pacing Arrhythm Card Electrophysiol J Work Groups Card Pacing Arrhythm Card Cell Electrophysiol Eur Soc Cardiol. 2023 Feb 16;25(2):536–45.

20. Jackson T, Claridge S, Behar J, Sieniewicz B, Gould J, Porter B, et al. Differential effect with septal and apical RV pacing on ventricular activation in patients with left bundle branch block assessed by non-invasive electrical imaging and in silico modelling. J Interv Card Electrophysiol Int J Arrhythm Pacing. 2020 Jan;57(1):115–23.

21. Nguyen UC, Stoks J, Cluitmans MJ, Strik M, van Rees B, Meiburg R, et al. Delayed ventricular conduction alters ventricular repolarization in patients with heart failure: an electrocardiographic imaging study. Heart Rhythm 2023 May 19-21 2023 New Orleans La. 2023 May 1;20(5, Supplement):S156–7.

22. Vijayakumar R, Silva JNA, Desouza KA, Abraham RL, Strom M, Sacher F, et al. Electrophysiologic substrate in congenital long QT syndrome: Noninvasive mapping with electrocardiographic imaging (ECGI). Circulation. 2014;130(22):1936–43.

23. Joy G, Lopes LR, Webber M, Ardissino AM, Wilson J, Chan F, et al. Electrophysiological Characterization of Subclinical and Overt Hypertrophic Cardiomyopathy by Magnetic Resonance Imaging-Guided Electrocardiography. J Am Coll Cardiol. 2024 Mar 19;83(11):1042–55.

24. Ramanathan C, Jia P, Ghanem R, Ryu K, Rudy Y. Activation and repolarization of the normal human heart under complete physiological conditions. Proc Natl Acad Sci. 2006 Apr 18;103(16):6309–14.
